# Supplementary material for: A quasi-randomised, controlled, feasibility trial of GLITtER (Green Light Imaging Interpretation to Enhance Recovery)—a psychoeducational intervention for adults with low back pain attending secondary care
Source: PeerJ. 2018 Feb 1;6:e4301. doi: 10.7717/peerj.4301 (PMC5797685; doi:10.7717/peerj.4301)
Supplement: Supplemental Information 1 [file peerj-06-4301-s001.pdf]

Supplement 1– Trial registration data

Data Category	Information
Primary registry and trial identifying number	ANZCTR:
Date of registration in primary registry	February, 2017
Secondary identifying numbers	Not applicable
Source(s) of monetary or material support	Royal Adelaide Hospital Research Foundation; NHMRC
Primary sponsor	Royal Adelaide Hospital Research Foundation
Secondary sponsor(s)	University of South Australia
Contact for public queries	EK, Emma.Karran@mymail.unisa.edu.au
Contact for scientific queries	EK, Emma.Karran@mymail.unisa.edu.au; LM, Lorimer.Moseley@unisa.edu.au
Public title	GLITtER (Green Light Imaging Interpretation to Enhance Recovery): a psychoeducational intervention for adults with low back pain attending secondary care
Scientific title	A quasi-randomised, controlled, feasibility trial of GLITtER (Green Light Imaging Interpretation to Enhance Recovery) – a psychoeducational intervention for adults with low back pain attending secondary care
Countries of recruitment	Australia
Health condition(s) or problem(s) studied	Low back pain; Chronic low back pain
Intervention(s)	Intervention group: Integration of the GLITtER intervention into 'standard practice' consultation
	Control group: 'Standard practice' consultation
Key inclusion and exclusion criteria	Ages eligible for study: ≥18 years; Sexes eligible for study: all; Accepts healthy volunteers: no
	Inclusion criteria: adult patient (18-65 years), attending Spinal Assessment Clinic, low back pain (+/- leg symptoms), access to a lumbar CT or MRI scan (performed during the previous 6 months)

	Exclusion criteria: unable to speak and understand English, history of lumbar spine surgery
Study type	Quasi-randomised, controlled, feasibility trial
Data of first enrolment	March 2 2017
Target sample size	40 (20 intervention, 20 control)
Recruitment status	Will commence March 2017
Primary outcomes(s)	Determine the feasibility of recruitment and retention, assessment procedures, implementation and acceptability of the "GLITtER' intervention
Key secondary outcomes	Identify any modifications needed in the design of a larger effectiveness trial.
	Provide data (i.e. the standard deviation of the primary outcome measure: numeric rating scale score for pain at 3 month follow-up) to permit calculation of an appropriately powered sample for a subsequent randomised controlled trial
