# Supplementary material for: A quasi-randomised, controlled, feasibility trial of GLITtER (Green Light Imaging Interpretation to Enhance Recovery)—a psychoeducational intervention for adults with low back pain attending secondary care
Source: PeerJ. 2018 Feb 1;6:e4301. doi: 10.7717/peerj.4301 (PMC5797685; doi:10.7717/peerj.4301)

GLITtER: Green Light Imaging Interpretation to Enhance Recovery

A framework for content delivery for SAC Clinicians

Emma Karran

OVERVIEW OF THE 'GLITTER' CONSULTATION:

- Routine SAC Assessment
- Completion of Eligibility Checklist (by SAC clinician)
- Routine management + GLITTER Consultation (Integrated)

Glitter Consultation Includes:

- PART 1: Review of Imaging (incorporate 'GREEN LIGHT' interpretation)
- PART 2: Management Recommendations (incorporate activity TICK list)
- PART 3: Introduction to take-home information (weeks 1-4)
- PART 4: Completion of GLITTER Checklist (by patient)
- PART 5: Provision of information regarding follow-up (on the back of the GLITTER checklist)

Communication with GP:

- PART 6: GLITTER addition to standard reporting template

Eligibility Checklist (SAC Clinician to complete following patient assessment)

UR Number: ______________

Does this patient…..

1.	Require further imaging, investigations or intervention?	Yes	No
2.	Require further surgical opinion?	Yes	No
3.	Have lumbar pathology warranting significant caution with activity?	Yes	No
4.	Engage in their usual activity, unrestricted by pain?	Yes	No
5.	Engage in regular exercise, unrestricted by pain?	Yes	No

(Circle)

- Eligibility confirmed if "NO" for ALL questions

PART 1: Review of imaging (incorporate 'GREEN LIGHT' interpretation)

E.g. Let's have a look at your scans (put scans in view…..)

Before we have a close look I want to explain to you a bit about normal findings on scans.

- Aim: **Re-frame normal findings** (prior to interpreting the patient's scans)
  - Refer to graph: findings in *asymptomatic* adults

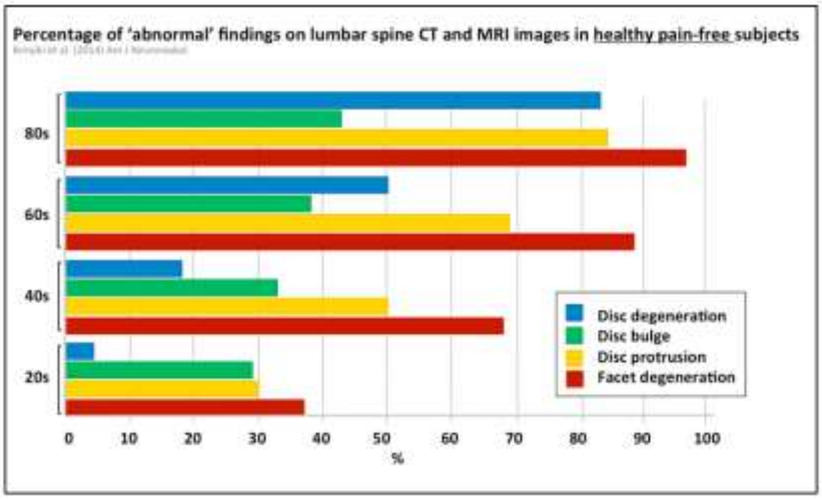

  - We now understand that many scan findings like degeneration and disc bulges are actually completely normal – you can think of them a bit like wrinkly skin or grey hair: as physical changes that 'just happen' over time.

‘Walk through’ patient scan results. (Discuss this further with YH).

   - Include orientation, explanation and interpretation of findings.

Highlight the following points, using visual aids as appropriate:

- Most/many of the findings on your scan are also found on scans of people who have never had back pain.
   - They would have been present well BEFORE your back pain started.
   - And they will still be there once your back pain has settled.
   - Visual aid: Refer to 2 images from PAINFREE individuals (not sure – to discuss)

- There is also often very little link between scan findings and a person’s activity levels
   - Visual aid (optional): Show tennis player or older jogger (depending on age) and their matched scans.

- Summary: Scans (on their own) actually don’t explain much about
   - Your current pain – especially why you have good days and bad
   - The activity you are capable of
   - Or how likely you are to recover: because the changes on your scans will still be there when your pain goes away.

Refer back to scans:

- “I want to show you a few things that I see when I look at your scans……..”
(Reinforce that spine is structurally sound and needs to move – via evidence on scans)

For example -

- Evenly sized vertebrae that are well aligned
- A spine that has adapted to load over time….
   - Thickening around joints and ligaments, narrowing and bulging of discs….these are not abnormal – they are like the wrinkles in your skin that deepen as you age
- A structure that is really strong, and withstands all sorts of stresses and strains. It has a crucial role of protecting the spinal cord and nerves throughout your life which is why its strength is so important.
   - Supported at the front and back by strong ligaments
   - Vertebrae connected by discs and facet joints designed to give stability
   - Big areas of muscle bulk on the front and back
- A spine that (as well as being stable and strong) **<u>needs movement</u> to be healthy**

It is safe for you to be active and to increase your activity levels, and it is important for your recovery that you do so.

(“you have been given the green light”: if it works to toss in this phrase or reference to “green light” then do so**)**

PART 2: Management recommendations (incorporate activity TICK list)

Offer usual management recommendations.

In addition, introduce to the TICK list (suggest it as a strategy that they might like to use to help with scheduling some extra activity or exercise into their week).

PART 3: Introduce to further information (weeks 1-4), provide magnets for fridge!

- Explain that they will be given the poster to take home which has 4 weeks-worth of information. The will put up 1 week at a time, and will be sent a text message each week to remind them to change it. They will also be sent a link to some further online information about pain, recovery and exercise.
- The information includes:
  - A re-cap of things we have spoken about today regarding your scans.
  - Some interesting things about PAIN and how complex it is. Pain doesn't always mean that you are doing damage to yourself.
  - The importance of activity and exercise for your recovery
  - And gives you a weekly TICK list to fill in at the start of each week – which might help you to get started with moving more.

PART 4: GLITTER Checklist (<u>patient to complete</u> at end of the session)

After my appointment today I understand that……..

1. I need an operation on my back	Yes	Possibly	No
2. I need further scans of my back	Yes	Possibly	No
3. My scans show things I should worry about	Yes	Possibly	No
4. It is safe to be active and exercise	No	Unsure	Yes
5. Activity and exercise will help my recovery	No	Unsure	Yes

(circle the answer you agree with)

PART 5: Provide with information regarding follow-up (on the back of the GLITTER checklist):

WHAT TO EXPECT following your appointment today:

- You will receive a brief, follow up text message next Monday. You do not need to reply to this message.
- You will receive 3 further brief text messages on the following 3 Mondays. (No reply required).
- 1 month after this appointment: you will receive some forms in the mail to fill in and return (in a postage-paid envelope).
- 3 months after this appointment: you will receive some further forms to fill in and return.
- You will not be contacted further after we have received your completed follow-up forms.
- Results of this study will be able to be found at: www.bodyinmind.org

THANK YOU very much!

PART 6: Communication with GP - GLITTER reporting template

Your patient has been included in a pilot study being conducted in the Spinal Assessment Clinic. This study is designed to assess the feasibility of integrating a novel educational intervention into the standard clinic consultation.

The intervention involves:

- *Explanation of 'normal' imaging findings and interpretation of the patient's images*
- *Provision of take-home information*
- *Weekly follow-up SMS messages (for 4 weeks) with links to online resources*
- *Provision of a practical tool to facilitate increased activity/exercise*

The intervention aims to deliver a 'positive' interpretation of imaging findings to reassure patients that movement is safe and is necessary for recovery. Key messages are:

- *Surgical intervention is not indicated and further scans are not required*
- *Scan findings should not cause worry; it is safe to be active*
- *Pain is complex (chronic pain often has little to do with damage)*
- *Activity and exercise are important for recovery and have many benefits*

Further information can be obtained by contacting the Principal Investigator:
Emma.Karran@mymai.unisa.edu.au

Figure 1. Study Flow: Prospective, comparative, feasibility trial of GLITTER

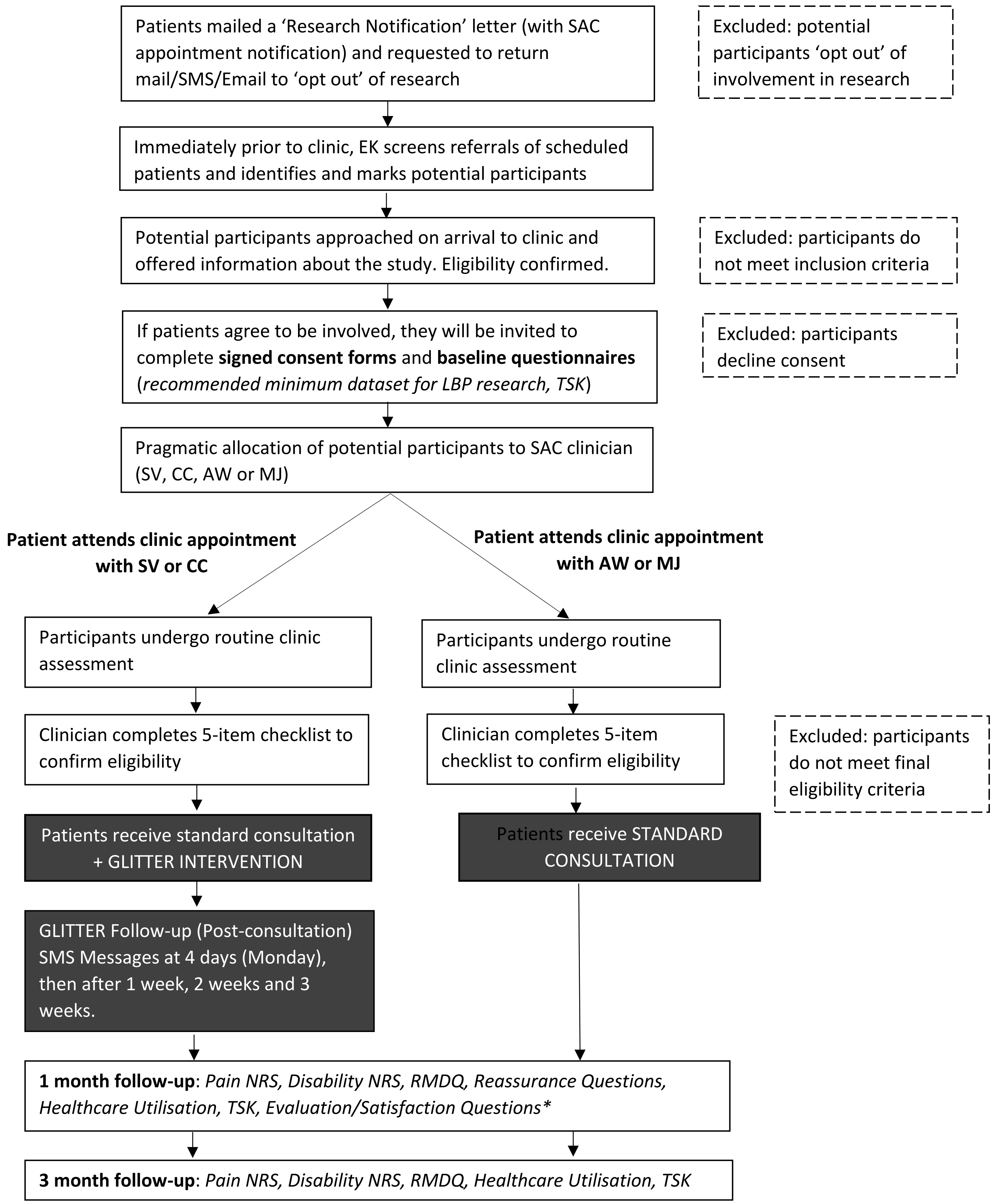

Supplement: Supplemental Information 3 [file peerj-06-4301-s003.pdf]
