# Supplementary material for: A quasi-randomised, controlled, feasibility trial of GLITtER (Green Light Imaging Interpretation to Enhance Recovery)—a psychoeducational intervention for adults with low back pain attending secondary care
Source: PeerJ. 2018 Feb 1;6:e4301. doi: 10.7717/peerj.4301 (PMC5797685; doi:10.7717/peerj.4301)
Supplement: Supplemental Information 4 [file peerj-06-4301-s004.pdf]

Supplement 4 – Outcome measures (1): Pain and Disability NRS & Reassurance Questions

---

1. In the past 7 days, how would you rate the **intensity of your pain** on average? (Circle a number)

0	1	2	3	4	5	6	7	8	9	10
**No pain**										**Worst pain imaginable**

2. In the past 7 days, how much has your pain **interfered with your daily activities** on average? (Circle a number)

0	1	2	3	4	5	6	7	8	9	10
**No interference**										**Unable to carry on activities**

3. Rate your belief in how likely it is that you will require an operation on your back? (circle a number)

0	1	2	3	4	5	6	7	8	9	10
**Not at all likely**										**Extremely likely**

4. How reassured are you that there is nothing seriously wrong with your back? (circle a number)

0	1	2	3	4	5	6	7	8	9	10
**Extremely reassured**										**Not at all reassured**

5. Do you think more tests or scans are needed at this stage? (circle) **Y / N**
