# Supplementary material for: A quasi-randomised, controlled, feasibility trial of GLITtER (Green Light Imaging Interpretation to Enhance Recovery)—a psychoeducational intervention for adults with low back pain attending secondary care
Source: PeerJ. 2018 Feb 1;6:e4301. doi: 10.7717/peerj.4301 (PMC5797685; doi:10.7717/peerj.4301)
Supplement: Supplemental Information 5 [file peerj-06-4301-s005.pdf]

Supplement 5 – Outcome measures (2): Participant Experience Questionnaire & Healthcare Utilisation Questions

---

Please rate your responses to the following statements by ticking the appropriate box:

	Strongly disagree	Disagree	Agree	Strongly agree
1. The clinician who saw me in the Spinal Clinic knew what they were talking about				
2. The clinician used simple, clear language that I could easily understand				
3. The information on the handout was relevant to me				
4. The information on the handout was helpful				
5. It was good to get the follow-up text messages				
6. Overall, I was satisfied with the care I received from the Spinal Clinic				
7. The information I have received from the Spinal Clinic will assist my recovery				

Healthcare use since your Spinal Clinic appointment:

1. In the past month, **have you seen a general practitioner** for your back pain?

   Y / N   If so, how many times? ____

2. In the past month, **have you seen a physiotherapist** for your back pain?

   Y / N   If so, how many times? ____

3. In the past month, **have you seen a chiropractor, massage therapist or other health care professional** for your back pain?

   Y / N   If so, how many times? ____
