# Supplementary material for: A quasi-randomised, controlled, feasibility trial of GLITtER (Green Light Imaging Interpretation to Enhance Recovery)—a psychoeducational intervention for adults with low back pain attending secondary care
Source: PeerJ. 2018 Feb 1;6:e4301. doi: 10.7717/peerj.4301 (PMC5797685; doi:10.7717/peerj.4301)
Supplement: Supplemental Information 6 [file peerj-06-4301-s006.pdf]

Supplement 6 - Recommended Minimum Dataset for Low Back Pain Research (Deyo et al. 2014): participant baseline data

**Item***<br>(Proportion of complete responses)	**N Total sample**					
1. How long has LBP been an ongoing problem for you? (97%)	Less than 1month<br>**0**	1-3months<br>**0**	3-6months<br>**0**	6months - 1year<br>**11**	1-5years<br>**5**	>5years<br>**11**
2. How often has LBP been an ongoing problem over the past 6 months? (97%)	Every day or nearly every day<br>**24**	At least half the days<br>**2**	Less than half the days<br>**4**			
4. Has back pain spread down your leg in the past 2 weeks? (97%)	Yes<br>**22**	No<br>**2**	Not sure<br>**3**			
5. During the past 4 weeks how much have you been bothered by…..<br>…..stomach pain? (87%)	Not bothered<br>**16**	Bothered a little<br>**9**	Bothered a lot<br>**2**			
…..pain in your arms, legs or joints (90%)	Not bothered<br>**3**	Bothered a little<br>**13**	Bothered a lot<br>**5**			
…..headaches (87%)	Not bothered<br>**9**	Bothered a little<br>**13**	Bothered a lot<br>**5**			
…..widespread pain in most of your body (94%)	Not bothered<br>**7**	Bothered a little<br>**12**	Bothered a lot<br>**10**			
6. Have you ever had a back operation? (100%)	Yes, one<br>**5**	Yes, > one<br>**0**	No<br>**26**			
In the past 7 days…..						
8. …..How much did pain interfere with work around the home? (100%)	Not at all<br>**0**	A little<br>**4**	Somewhat<br>**9**	Quite a bit<br>**11**	Very much<br>**7**	
9. ……How much did pain interfere with your ability to participate in social activities? (100%)	Not at all<br>**1**	A little<br>**6**	Somewhat<br>**7**	Quite a bit<br>**11**	Very much<br>**6**	
10. …..How much did pain interfere with your household chores? (100%)	Not at all<br>**0**	A little<br>**5**	Somewhat<br>**6**	Quite a bit<br>**13**	Very much<br>**7**	

11. Have you used any of the following treatments for your back pain? .....Opioid painkillers (100%)	Yes **19**	No **12**			
.....injections (100%)	Yes **15**	No **15**			
.....exercise therapy (84%)	Yes **14**	No **12**			
.....psychological counselling (94%)	Yes **2**	No **26**			
12. I have been off work or unemployed for 1 month or more due to LBP (94%)	Agree **10**	Disagree **4**	Does not apply **16**		
13. I receive or have applied for disability or workers compensation benefits because I am unable to work due to LBP (94%)	Agree **2**	Disagree **5**	Does not apply **23**		
14. Are you able to do chores such as vacuuming or yard work? (100%)	Without difficulty **3**	With a little difficulty **3**	With some difficulty **16**	With much difficulty **5**	Unable to do **4**
15. Are you able to go up and down stairs at a normal pace? (94%)	Without difficulty **2**	With a little difficulty **8**	With some difficulty **11**	With much difficulty **6**	Unable to do **3**
16. Are you able to go for a walk of at least 15 minutes? (94%)	Without difficulty **7**	With a little difficulty **6**	With some difficulty **9**	With much difficulty **5**	Unable to do **3**
17. Are you able to run errands and shop? (94%)	Without difficulty **7**	With a little difficulty **11**	With some difficulty **8**	With much difficulty **4**	Unable to do **0**
In the past 7 days…..					
18. …..I felt worthless (94%)	Never **13**	Rarely **3**	Sometimes **8**	Often **4**	Always **1**
19. …..I felt helpless (94%)	Never **10**	Rarely **4**	Sometimes **8**	Often **6**	Always **1**
20. …..I felt depressed (94%)	Never **9**	Rarely **5**	Sometimes **6**	Often **9**	Always **1**

21. …..I felt hopeless (97%)	Never<br>**10**	Rarely<br>**5**	Sometimes<br>**8**	Often<br>**6**	Always<br>**1**
22. …..my sleep quality was (100%)	Very poor<br>**8**	Poor<br>**13**	Fair<br>**9**	Good<br>**1**	Very good<br>**0**
23. …..my sleep was refreshing (94%)	Not at all<br>**8**	A little bit<br>**12**	Somewhat<br>**6**	Quite a bit<br>**3**	Very much<br>**0**
24. …..I had a problem with my sleep (94%)	Not at all<br>**1**	A little bit<br>**8**	Somewhat<br>**5**	Quite a bit<br>**8**	Very much<br>**7**
25. …..I had difficulty falling asleep (97%)	Not at all<br>**8**	A little bit<br>**5**	Somewhat<br>**6**	Quite a bit<br>**7**	Very much<br>**4**
26. It's not really safe for a person with my back problem to be physically active (100%)	Agree<br>**13**	Disagree<br>**18**			
27. I feel that my back pain is terrible and it's never going to get any better (97%)	Agree<br>**16**	Disagree<br>**14**			
28. Are you involved in a lawsuit or legal claim related to your back problem? (100%)	Yes<br>**0**	No<br>**30**	Not sure<br>**1**		
In the past year…..					
29. …..Have you ever been drunk or used drugs more than you meant to? (97%)	Never<br>**18**	Rarely<br>**6**	Sometimes<br>**5**	Often<br>**1**	
30. …..Have you felt you wanted or needed to cut down on your drinking or drug use? (87%)	Never<br>**21**	Rarely<br>**1**	Sometimes<br>**3**	Often<br>**2**	
32. Gender (100%)	Female<br>**19**	Male<br>**12**	Unknown<br>**0**	Unspecified<br>**0**	
33. Employment status (94%)	Working now<br>**7**	Looking for work, unemployed<br>**5**	Sick leave or maternity leave<br>**0**	Disabled due to back pain, permanently or temporarily<br>**4**	Disabled for reasons other than back pain<br>**1**
	Student<br>**1**	Temporarily laid off<br>**0**	Retired<br>**6**	Keeping house<br>**3**	Other, specify<br>**2** (1=carer, 1=unknown)

34.	Education level (97%)	Did not complete high school<br>**14**	Completed high school<br>**3**	Some further education, no degree<br>**5**	Occupational/ technical/ vocational program<br>**3**	Completed university degree<br>**4**	Completed university post-graduate qualification<br>**1**
35.	How would you describe your cigarette smoking? (97%)	Never smoked<br>**9**	Current smoker<br>**13**	Used to smoke but now quit<br>**8**			
36.	Body Mass Index (87%)	Underweight (BMI <18.5)<br>**1**	Healthy (BMI 18.5-24.9)<br>**10**	Overweight (BMI 25-29.9)<br>**6**	Obese (BMI 30+)<br>**10**		

*NRS Items 3 (pain intensity) and 7 (pain interference) are reported in the manuscript (Table 4). Item 31 (age) is reported in the results section of the manuscript

Deyo RA, Dworkin SF, Amtmann D, Andersson G, Borenstein D, Carragee E, Carrino J, Chou R, Cook K, DeLitto A, Goertz C, Khalsa P, Loeser J, Mackey S, Panagis J, Rainville J, Tosteson T, Turk D, Von Korff M, and Weiner DK. 2014. Report of the NIH Task Force on Research Standards for Chronic Low Back Pain. *The Journal of Pain* 15:569-585. http://dx.doi.org/10.1016/j.jpain.2014.03.005
