# Supplementary material for: A quasi-randomised, controlled, feasibility trial of GLITtER (Green Light Imaging Interpretation to Enhance Recovery)—a psychoeducational intervention for adults with low back pain attending secondary care
Source: PeerJ. 2018 Feb 1;6:e4301. doi: 10.7717/peerj.4301 (PMC5797685; doi:10.7717/peerj.4301)

Supplement 7 – Visual aid A

Percentage of 'abnormal' findings on lumbar CT and MRI images in pain-free subjects

Brinjikji et al. (2014) Am J Neuroradiology

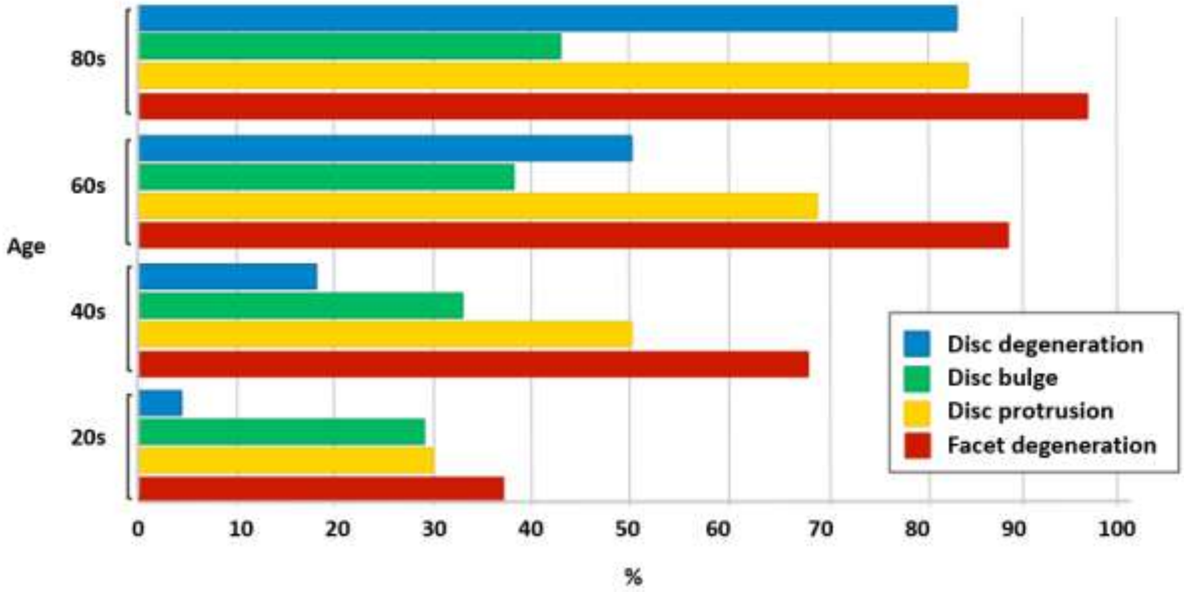

Supplement: Supplemental Information 7 [file peerj-06-4301-s007.pdf]
