# Supplementary material for: A quasi-randomised, controlled, feasibility trial of GLITtER (Green Light Imaging Interpretation to Enhance Recovery)—a psychoeducational intervention for adults with low back pain attending secondary care
Source: PeerJ. 2018 Feb 1;6:e4301. doi: 10.7717/peerj.4301 (PMC5797685; doi:10.7717/peerj.4301)
Supplement: Supplemental Information 8 [file peerj-06-4301-s008.pdf]

Supplement 8 – Take-home information resource

WEEK 1 — IMPORTANT THINGS TO REMEMBER AFTER YOUR APPOINTMENT

SCANS

DO NOT Tell us much about:

- The amount of pain you feel now
- Your likely pain in the future

DO SHOW Age-related changes:

- Many reported changes sound abnormal, but occur naturally with age
- For example: disc bulges and degeneration
- These are like "wrinkles on the inside"

DO SHOW Findings that:

- Would have been there before your pain began
- Will still be there when your pain has gone

DO SHOW The structure of your spine:

- It is strong, stable and surrounded by large muscles
- It has adapted to load over time
- It needs movement to be healthy

After reviewing **you and your scans** in the Spinal Clinic — You have been given the **green light** — It is **safe and necessary** to MOVE and EXERCISE

THIS WEEK: To learn more about recovering well from back pain

Plan **15 minutes** to watch .... **The truth about back pain** (we will SMS you a link)

or find it at: https://www.youtube.com/watch?v=b-cBtPSf0Hc

Doing regular exercise **or** becoming more physically active is important for your recovery.

HOW TO GET STARTED: Consider: ......

- What do you like to do?
- What have you enjoyed doing in the past?
- What would you like to do more of?
- What could you do regularly with a friend or family member?

WRITE IT ON YOUR ACTIVITY TICK LIST

PLAN TIME TO EXERCISE THIS WEEK

MAKE A **T**IME

IT'S **I**MPORTANT

BE **C**ONSISTENT

KNOW YOUR BACK IS UP TO IT

WHAT	WHEN	TICK
30 min walk with Sue	Monday 7pm	✓

WEEK 2

DO YOU KNOW HOW COMPLEX PAIN IS?

RECOVERY ROADS: which road are you on?

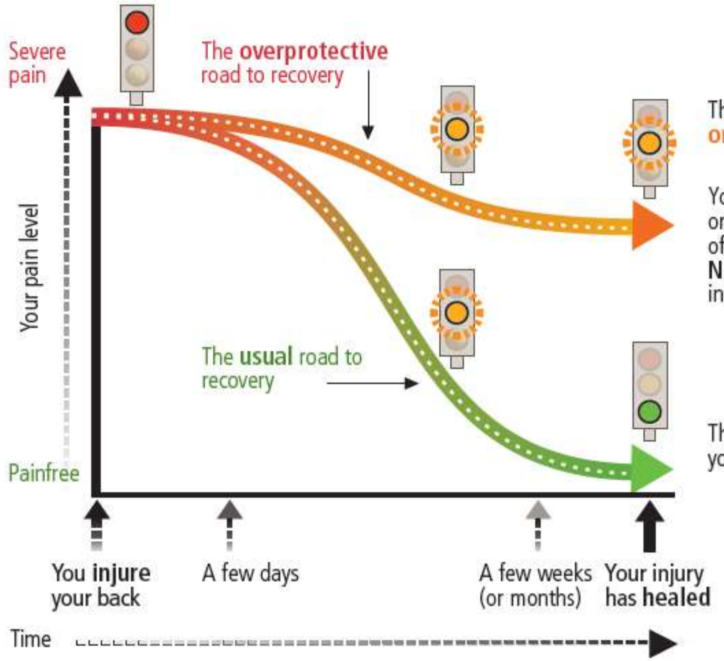

The light is still flashing **orange**.

Your pain and its impact on your life are both the result of an **over-protective** system, **NOT** an ongoing injury in your back.

The light is **green** and you feel safe to move.

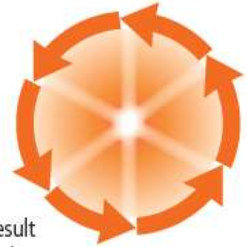

MANY THINGS

can make your system **over-protective**

- Painful experiences with movement
- Stress
- Being worried about your back
- Lack of movement
- Low mood
- Poor general health

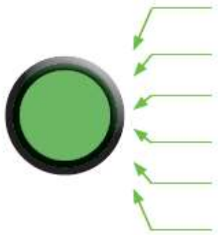

HELP THE LIGHT TURN GREEN

by looking after yourself well

- Sleeping well
- Eating well
- Reducing stress
- Increasing activity
- Relaxing
- Socialising

PLAN TIME TO EXERCISE THIS WEEK

THIS WEEK: Learning more about PAIN can help your recovery.

PLAN 5 MINUTES to watch ... Tame the Beast
FIND IT AT: https://www.youtube.com/watch?v=XwBYkw-iZdQ

MAKE A **T**IME
IT'S **I**MPORTANT
BE **C**ONSISTENT
KNOW YOUR BACK IS UP TO IT

WHAT	WHEN	TICK

WEEK 3 EXERCISE IS BENEFICIAL FOR...

The health of your back:
Increases strength, endurance and flexibility.
Your back needs to move!

Your psychological wellbeing:
Improves mood, reduces stress and promotes better sleep. It can be enjoyable and social.

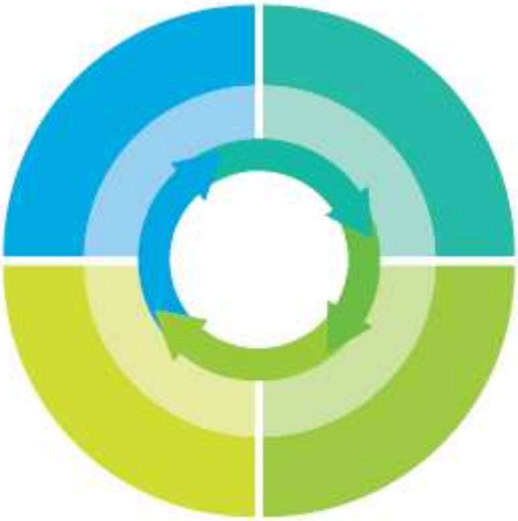

Your general health:
Regular exercise can help you to do things you want and need to do, and helps with weight control.

Reducing risks for other health problems:
Helps to prevent and manage conditions such as heart disease, diabetes and cancer.

REMEMBER: PAIN IS COMPLEX
When pain is produced by an overprotective system, it has little to do with damage

Knowing this means:

- You can worry less when you feel pain
- You can continue moving, even when you are in pain
- You can gradually do more of the active things that you enjoy

EXERCISE can also help to correct your over-protective signalling:

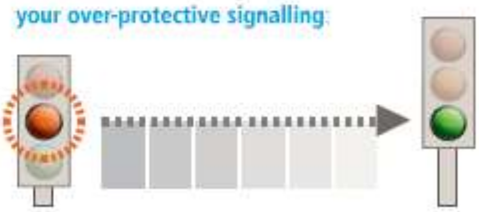

THIS WEEK: If being active isn't an enjoyable part of your everyday life -
Spend 10 minutes looking at: 'How to Start Exercising and Stick to It' THEN....... make a plan!
FIND IT AT: https://www.helpguide.org/articles/healthy-living/how-to-start-exercising-and-stick-to-it.htm

WRITE IT ON YOUR **ACTIVITY TICK LIST**
PLAN **TIME** TO EXERCISE THIS WEEK

MAKE A **T**IME
IT'S **I**MPORTANT
BE **C**ONSISTENT
KNOW YOUR BACK **I**S UP TO **I**T

WHAT	WHEN	TICK

Cite this as: Karran et al. 2017

WEEK 4

5 IMPORTANT THINGS TO REMEMBER

WHAT DO YOU LIKE TO DO ?

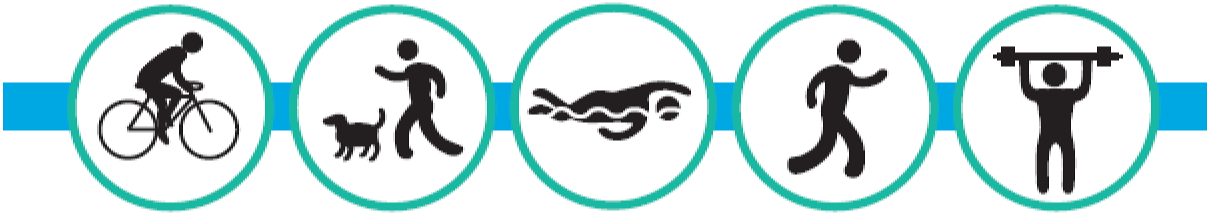

1 Your back needs movement to be healthy

2 You were given the green light: movement and activity is safe

3 Many things influence pain – knowing this can help you worry less about your back

4 Long-lasting back pain often has little to do with damage

5 Regular exercise is important for your recovery and has many benefits

If you want to understand more about pain and what you can do about it plan 5 minutes to watch....... **Understanding pain in less than 5 minutes**

FIND IT AT: https://www.youtube.com/embed/qEWc2XtaNwg

OVER THE WEEKS AHEAD:

- Continue to make PLANS for exercise – it is **IMPORTANT**.
- Be **HOPEFUL** that you will return to most of the things you like to do.
- **EXPECT** to have 'ups and downs': life has ups and downs.
- Talk to your Doctor or Physio if you have concerns (and take this sheet with you!)

WRITE IT ON YOUR ACTIVITY TICK LIST

PLAN **TIME** TO EXERCISE THIS WEEK

MAKE A **T**IME
IT'S **I**MPORTANT
BE **C**ONSISTENT
KNOW YOUR BACK IS UP TO IT

WHAT	WHEN	TICK

Cite this as: Karran et al. 2017
