# Supplementary material for: A quasi-randomised, controlled, feasibility trial of GLITtER (Green Light Imaging Interpretation to Enhance Recovery)—a psychoeducational intervention for adults with low back pain attending secondary care
Source: PeerJ. 2018 Feb 1;6:e4301. doi: 10.7717/peerj.4301 (PMC5797685; doi:10.7717/peerj.4301)
Supplement: Supplemental Information 9 [file peerj-06-4301-s009.pdf]

Supplement 9: Letter to General Practitioner

Your patient has been included in a pilot study being conducted in the Spinal Assessment Clinic. This study is designed to assess the feasibility of integrating a novel educational intervention into the standard clinic consultation.

The intervention involves:

- *Explanation of 'normal' imaging findings and interpretation of the patient's images*
- *Provision of take-home information*
- *Weekly follow-up SMS messages (for 4 weeks) with links to online resources*
- *Provision of a practical tool to facilitate increased activity/exercise*

The intervention aims to deliver a 'positive' interpretation of imaging findings to reassure patients that movement is safe and is necessary for recovery. Key messages are:

- *Surgical intervention is not indicated and further scans are not required*
- *Scan findings should not cause worry; it is safe to be active*
- *Pain is complex (chronic pain often has little to do with damage)*
- *Activity and exercise are important for recovery and have many benefits*

Further information can be obtained by contacting the Principal Investigator:
Emma.Karran@mymai.unisa.edu.au
