# Supplementary material for: A quasi-randomised, controlled, feasibility trial of GLITtER (Green Light Imaging Interpretation to Enhance Recovery)—a psychoeducational intervention for adults with low back pain attending secondary care
Source: PeerJ. 2018 Feb 1;6:e4301. doi: 10.7717/peerj.4301 (PMC5797685; doi:10.7717/peerj.4301)
Supplement: Supplemental Information 10 [file peerj-06-4301-s010.pdf]

Supplement 10 – Final patient eligibility checklist

(Completed by clinician. Clinician must circle all "No" responses for patient to meet final eligibility criteria.)

Does this patient…..

1. Require further imaging, investigations or intervention?	Yes	No
2. Require further surgical opinion?	Yes	No
3. Have lumbar pathology warranting significant caution with activity?	Yes	No
4. Engage in their usual activity, unrestricted due to pain?	Yes	No
5. Engage in regular exercise, unrestricted due to pain?	Yes	No

Clinician: ______________________
