# Supplementary material for: A quasi-randomised, controlled, feasibility trial of GLITtER (Green Light Imaging Interpretation to Enhance Recovery)—a psychoeducational intervention for adults with low back pain attending secondary care
Source: PeerJ. 2018 Feb 1;6:e4301. doi: 10.7717/peerj.4301 (PMC5797685; doi:10.7717/peerj.4301)
Supplement: Supplemental Information 11 [file peerj-06-4301-s011.pdf]

Supplement 11 – Trial protocol

A quasi-randomised, controlled, feasibility trial of GLITtER (Green Light Imaging Interpretation to Enhance Recovery) – a psychoeducational intervention for adults with low back pain attending secondary care.

Reported in accordance with the SPIRIT 2013 Statement[1] and published on Open Science Framework (https://osf.io/8zrq3/)

Section 1. Administrative Information

Title
A quasi-randomised, controlled, feasibility trial of GLITtER (Green Light Imaging Interpretation to Enhance Recovery) – a psychoeducational intervention for adults with low back pain attending secondary care.

Trial Registration
This trial will be registered on the ANZCTR prior to commencement (www.anzctr.org.au). Trial registration will include details of all items from the World Health Organisation Trial Registration Data Set (See Table 1 at end of Protocol).

Protocol Version
Issue date: 15 Dec, 2016, original
Authors: EK, LM

This protocol has been developed in accordance with the ***SPIRIT 2013 Checklist[1]*** (Standard Protocol Items: Recommendations for Interventional Trials) (see www.spirit-statement.org).

Funding
The principal researcher, Emma Karran receives funding from the Royal Adelaide Hospital Research Foundation Clinical Research Grant (2015) and the Royal Adelaide Hospital Research Foundation Dawes Scholarship.
Lorimer Moseley is supported by NHMRC (ID: 106279)
James McAuley is supported by NHMRC (ID: 1047827)

Roles and Responsibilities
Protocol contributors:
Emma L. Karran[1, 2]
Susan L. Hillier[1]
Yun-Hom Yau[2]
Lorimer Moseley[1]

Affiliations:
[1]School of Health Sciences, University of South Australia, Adelaide, SA
[2]Royal Adelaide Hospital, Adelaide, SA

Roles:

EK (together with YHY and LM) conceived the idea and designed the study. EK led the development of the GLITtER intervention. EK will continue to collaborate with clinical and administrative personnel, lead staff training, and recruit participants. She will be responsible for all participant follow-up, data collection, data analysis and interpretation of the results. EK will draft and revise reports and manuscripts arising from this study.
SH made contributions to study planning. She will assist with interpretation of the results and will be involved in the drafting and review of reports/manuscripts
YHY made substantial contributions to study conception and planning and was consulted in the development of the GLITtER intervention. YHY will assist with staff training and clinical implementation and will be involved in the review process for manuscripts arising from this study.
LM made substantial contributions to study conception and planning, and assisted with the development of the GLITtER intervention. GLM will be involved with the interpretation of data, and the drafting/revision of manuscripts for publication.

Principal researcher for correspondence:
Emma Karran, Emma.Karran@mymail.unisa.edu.au

Role of study sponsors and funders:
The funders (Royal Adelaide Hospital Research Foundation and NHMRC) played no role in the design of this study. They will have no role in the future collection, management, analysis or interpretation of data; writing of the report; or decision to publish.

Section 2. Introduction

Background and Rationale
Current practice guidelines universally recommend 'reassurance' as an important component of low back pain (LBP) management[2]. However, despite the recognition that clinicians are able to effectively reassure LBP patients (and with lasting effect),[3] how best to reassure – and its impact on pragmatic outcomes – is poorly understood.

Approximately 2000 adults with spinal pain are referred by medical practitioners (annually) for surgical opinion in the spinal outpatient clinics at the Royal Adelaide Hospital (RAH). Surgery, however, is likely to benefit less than 10% of these patients. Clinic consultations routinely involve review and explanation of each patient's imaging results – an issue to which the principal researchers of this study have given much consideration, in collaboration with clinical colleagues.

It has been suggested that the communication of imaging reports may increase fear of re-injury and reduce the likelihood of a good outcome[4]. Indeed, the highly prevalent 'abnormalities' detailed in radiology reports are rarely reassuring. Adverse effects of early imaging have been reported.[5, 6] Recent evidence suggests that many 'degenerative' changes found on spinal imaging are *not* abnormal and highly likely to be found in asymptomatic individuals.[7] It is also understood that spinal imaging findings are not well associated with pain or prognosis.[8, 9] Our intervention involves a new and standardised method of reporting radiological findings in a manner designed to reassure patients, and promote engagement in an active recovery.

A feasibility trial, conducted in the spinal outpatient setting at the RAH, is the crucial first step towards definitive testing of GLITtER (Green Light Imaging Intervention to Enhance Recovery).

Aim

The aim of this study is to inform feasibility of definitive testing of the GLITtER intervention in a randomised controlled trial. This trial would ultimately address the question of whether the proposed intervention – integrated into routine practice in a spinal outpatient clinic setting - is a cost-effective strategy for reducing chronic LBP and disability.

Objectives
The primary feasibility objectives are to:
- Determine the feasibility of recruitment and retention, assessment procedures, implementation and acceptability of the "GLITtER' intervention for LBP patients attending the Spinal Assessment Clinic (SAC).

Secondary objectives are to:
- Identify any modifications needed in the design of a larger effectiveness trial.
- Provide data (i.e. the standard deviation of the outcome measure) to permit calculation of an appropriately powered sample for a subsequent randomised controlled trial (RCT).

Study Design
This study is a *prospective, quasi-randomised feasibility trial* with longitudinal follow-up, involving sampling of patients scheduled on spinal outpatient clinic waiting lists.

This study will also adopt an *adaptive trial design,* which will allow modifications to be made during its conduct with the purpose of increasing the probability of success of the study procedure or the intervention. Any adaptations required to recruitment/study procedures or the GLITtER intervention will be made during recruitment of the first one-third of participants.

Section 3. Methods: Participants, Interventions and Outcomes

Study Setting
This study will take place in the Spinal Outpatient Clinic at the Royal Adelaide Hospital, Adelaide, SA, Australia. Spinal clinics operate from 9:30am until 1:00pm on Thursdays. Participants attending the Spinal Assessment Clinic (SAC) will be eligible for inclusion. The SAC is a Physiotherapist-led clinic attended by patients who warrant 'non-urgent' consultation as previously identified by a paper-based triage procedure.

Eligibility Criteria
Inclusion criteria :
- Adults with lumbar spine disorders (low back pain with or without leg symptoms)
- 18 – 75 year old men and women
- Paper-based triage undertaken and patients identified as "non-urgent", requiring scheduling on an outpatient clinic booking queue
- Patients have access to a recent CT or MRI scan of their lumbar spine (performed during the previous 6 months)

Exclusion criteria:
- Unable to speak and understand (verbal and written) English
- History of lumbar spine surgery

Interventions
Intervention Group:
The intervention group will receive 'GLITtER', integrated into the standard SAC consultation.
What is GLITtER (Green Light Imaging Interpretation to Enhance Recovery)?

- GLITtER is a brief intervention that will be integrated into current practice in the SAC
- It will extend the clinic consultation time by a maximum of 10 minutes
- GLITtER presents a framework for interpreting imaging findings
- Patients are given a metaphorical 'green light' – indicating that movement and activity is safe (and required)
- GLITtER offers a simple strategy for promotion of activity
- GLITtER offers take home information: designed as a 4-week series of key messages displayed in poster style.
- Involves SMS follow ups (X4) with links to online education resources
- Includes brief written communication with the patient's General Practitioner.

The GLITtER intervention will be described in accordance with the ***TIDieR*** (Template for Intervention Description and Replication) checklist (see www.equator-network.org). The TIDier checklist has been recommended to be used in conjunction with the SPIRIT statement as an extension of Item 11.

Control Group:
Participants allocated to the control group will receive 'standard care' in the SAC. This involves routine assessment, interpretation of imaging findings and advice regarding conservative management. Standard care will be delivered by clinicians who are naïve to the content of 'GLITtER' and operate according to what is considered to be best-practice SAC care. The components of GLITtER that are underlined above will be unique to the intervention group.

Outcomes
Primary Outcomes:
This study aims to determine the feasibility of recruitment and retention, assessment procedures, implementation and acceptability of the "GLITtER' intervention for LBP patients attending the Spinal Assessment Clinic (SAC).

Specific feasibility criteria are:
1. One subject per clinician per week can be recruited (4 Clinicians at each weekly clinic).
2. At least 70% of all eligible patients can be recruited.
3. Complete follow-up data are obtained in at least 95% of all recruited subjects.
4. GLITtER consultations do not extend clinic appointments by longer than 10 minutes (on average).
5. Acceptability of the "GLITtER' intervention for LBP patients attending the Spinal Assessment Clinic (SAC) is indicated by: >80% of responses to questions 1-7 of the Participant Experience Questionnaire are rated as "agree" or "strongly agree".
6. SAC Clinicians delivering GLITtER report that they are "confident" or "very confident" when asked: "How confident are you that you could integrate GLITtER into standard practice on an ongoing basis?", and "How confident are you that integrating GLITtER would enhance SAC care?" (4 point scale).

Secondary outcomes:
- To identify any modifications needed in the design of a larger effectiveness trial.
- Provide data (i.e. the standard deviation of the primary outcome measure: numeric rating scale score for pain at 3-month follow-up) to permit calculation of an appropriately powered sample for a subsequent randomised controlled trial (RCT).
- Exploratory analysis of changes in pain, disability, health care utilization and kinaesiophobia from baseline to follow-up will be assessed, however treatment effects will be interpreted with caution. Results will also be viewed for ceiling or floor effects.

The planned primary and secondary outcome measures of the intervention trial (and the time-point of assessment) are indicated in Table 2.

Table 2. Planned outcome measures and time points

Baseline	1 month	3 months
**Minimum dataset recommended for LBP research. Includes: Pain NRS, Disability NRS**	Pain NRS<br>Disability NRS	Pain NRS<br>Disability NRS
		RMDQ
-	Reassurance Questions	-
-	Healthcare Utilisation	Healthcare Utilisation
**Tampa Scale for Kinaesiophobia (TSK)**	TSK	TSK
-	Participant Experience Questionnaire	-

Participant Timeline
See Figure 1

Sample Size
40 participants will be recruited and allocated to intervention or control groups using a quasi-randomised procedure. This sample size is considered adequate for a feasibility study (designed principally to assess feasibility of recruitment and procedures), and will provide information enabling a power analysis for a subsequent randomised controlled trial.[10] The investigators acknowledge that estimates of treatment effect arising from this study are likely to be imprecise, due to the non-randomised allocation procedure and small sample size.

Recruitment
Potential participants will be mailed a 'Research Notification' letter and will be asked to return mail/SMS/Email if they wish to 'opt out' of involvement in research. If no 'opt out' information is received, potential participants will be approached in the waiting room when attending their spinal clinic appointment.

Procedure (see also Figure 1)

1. Patients attending the SAC will be mailed a 'Research Notification' letter (with their appointment notification letter) and will be asked to return mail/SMS/Email if they wish to 'opt out' of involvement in research. Patients who have opted out will not be approached for participation when they attend their clinic appointment.
2. Immediately prior to each clinic, EK will screen the referrals of all scheduled patients, and will identify and mark potential participants.
3. Potential participants will be approached on arrival to clinic and offered information about the study.
4. If patients agree to be involved, they will be invited to complete signed consent forms and baseline questionnaires (2).
5. All participants undergo routine clinic assessment.
6. At completion of the assessment, the clinician completes a 5-item checklist to confirm eligibility for GLITtER study:

Does this patient:

1. Require further imaging, investigations or intervention?	Yes / No
2. Require further surgical opinion?	Yes / No
3. Have lumbar pathology warranting significant caution with activity?	Yes / No
4. Engage in their usual activity, unrestricted due to pain?	Yes / No
5. Engage in regular exercise, unrestricted due to pain?	Yes / No

To be eligible for inclusion, all items must be answered "No".

7. If the participant meets final eligibility criteria: patients who are attending an appointment with AW or MJ will receive a 'standard' consultation. Patients attending an appointment with SV or CC will receive a GLITtER consultation.
8. AW & MJ will complete eligibility checklist for all patients, until 20 participants have been included in the study (as control participants). Participants will be notified of their inclusion and provided an information sheet to inform them that they will receive forms 1 month and 3 months post appointment for completion, along with SMS reminders.
9. SV & CC will complete the checklist until one patient (each) per clinic has been identified as eligible and one GLITtER consultation has been carried out. This will continue for 10 weeks until 20 patients have received the GLITtER intervention.

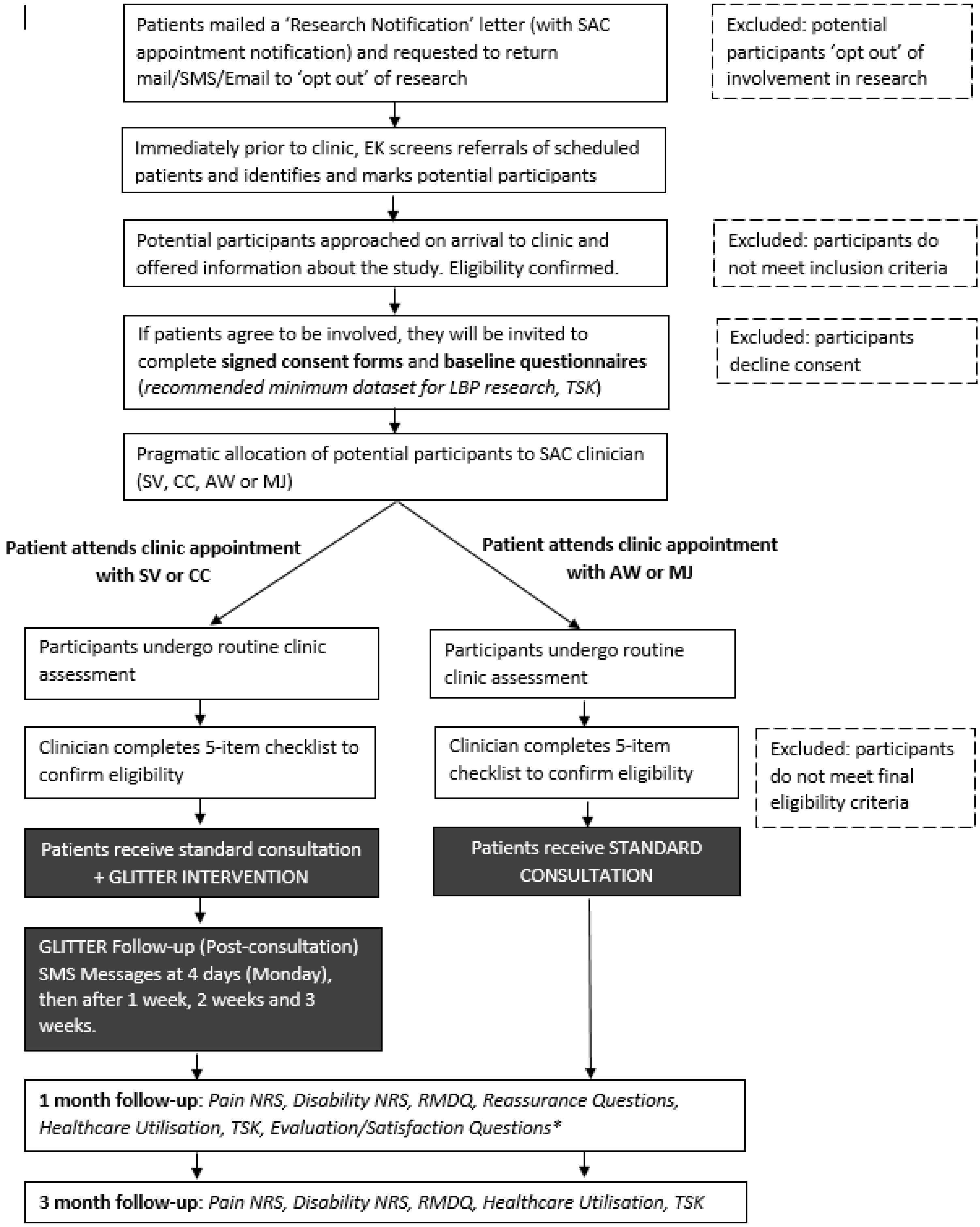

Figure 1. Study Flow: Prospective, comparative, feasibility trial of GLITTER

Section 4. Methods: Assignment of Interventions

Allocation

Participants will be allocated to the GLITtER Intervention or the control group via a quasi-randomised procedure that will cause minimal interference to clinic processes and also allow between-group comparisons. Two clinicians (SV and CC) are currently aware of the content of GLITtER and will require minimal training to be adequately skilled to be able to integrate GLITtER into their routine consultation. Two clinicians (AW and MJ) are (and will remain) naïve to the GLITtER content. The control group will therefore receive care which is considered representative of 'standard practice'. Group allocation will be determined by appointment scheduling and clinician availability.

Blinding

Trial participants will be blinded (they will be unaware whether they received a GLITtER consultation or a standard consultation).

Section 5. Methods: Data Collection, Management and Analysis

Data Collection

Collection of baseline and outcome data:

Baseline demographic and outcome data will be hand-recorded by participants on purpose-designed forms. Follow-up data will be complete either via postal questionnaires, telephone call or online questionnaires –depending on participant preference.

Description of instruments, including reliability and validity:

Data collection forms are included in the Appendices (3-9)

- Recommended Minimum Data Set for LBP Research[11] (Appendix 3) – Research standards were developed and published in 2014 by the National Institutes for Health Pain Consortium with recommendations for the minimum dataset to be collected when conducting low back pain research.
- Tampa Scale for Kinaesiophobia-11 (TSK-11)[12] (Appendix 4) – The TSK-11 has been demonstrated to be a brief, reliable, and valid measure of fear of movement/(re)injury for chronic pain patients.
- Pain Numeric Rating Scale (Pain NRS) (Appendix 5) - Pain NRS have been recommended for clinical and research use as a simple and adequate method of detecting changes in pain intensity among chronic pain patients.[13]
- Disability Numeric Rating Scale (Disability NRS) (Appendix 5) - NRS for functional impairment have been demonstrated to have satisfactory and similar properties to a multi-item functional scale in the evaluation of chronic musculoskeletal conditions.[14]
- Reassurance questions: 3 Questions (Appendix 5) – not validated
- Roland Morris Disability Questionnaire (RMDQ)[15] (Appendix 6) – The RMDQ is a widely implemented, back-specific, multi-item measure of physical function, with demonstrated reliability and validity.[16]
- Participant Experience Questions (Appendix 8) – not validated
- Health Care utilisation questions: 3 Questions (Appendix 7) – not validated

Data Management

The raw data will be entered onto purpose-designed forms and transferred onto a password-protected Excel spread sheet. Electronic and hard copies will be stored in a secure location for a minimum of 5 years.

Statistical Methods
Baseline clinical and demographic characteristics of the participants will be reported using descriptive statistics. Patient eligibility, recruitment and retention rates will be calculated, and reasons for refused consent will be recorded. Questionnaire completion rates will also be calculated. Exploratory analysis of changes in pain, disability and kinaesiophobia from baseline to follow-up will be assessed using an ANOVA, however treatment effects will be interpreted with caution. The standard deviation of the primary outcome measure (pain NRS at 3 months) will be used to inform future sample size calculations for a larger RCT.

Section 6. Methods: Monitoring

Data Monitoring – Interim Evaluation
Interim evaluation of the trial (led by the principal researcher) will occur after 1 month follow-up data has been received from 30% of participants. At this stage:

- Any issues/problems with participant recruitment procedures will be identified.
- Discussions will occur with SV and CC to seek feedback and concerns regarding
    a. Pilot study procedures
    b. Content of the intervention
    c. Any other issues
- Collected data will be assessed for completeness
- Participant Experience questionnaires will be reviewed for feedback regarding acceptability of the GLITtER intervention.

Issues identified will be discussed with the clinical and research team (as appropriate) and any modifications to the study protocol or the intervention will be made as required.

An external data monitoring committee is not needed since this is a low risk study and no data analysis will occur at the interim stage.

Harms
There are no anticipated risks or harms associated with this study. Any unanticipated adverse events will be documented and reported and managed appropriately.

Auditing
No auditing of the feasibility trial will occur.

Section 7. Ethics and Dissemination

Research Ethics Approval
This study has been approved by the Human Research Ethics Committees at the Royal Adelaide Hospital and the University of South Australia.

Protocol Amendments

Protocol modifications (prior to commencing the study or at interim stage) will be communicated with the human research ethics committees at the University of South Australia and the Royal Adelaide Hospital, as well as the clinical trials registry (ANZCTR).

Consent
The principal researcher (EK) will introduce the feasibility trial to potential participants and provide an information sheet. Discussion regarding participation will be invited, in light of the information received. Written consent will then be obtained from participants willing to be involved in the study.

Confidentiality
All study-related information will be stored securely in password-protected electronic format or in a locked filing cabinet at the study site. All participant information will be stored in a locked filing cabinet in an area with limited access. All records that contain names or other personal identifiers, such as locator forms and informed consent forms, will be stored separately from study records identified by code number. All local databases will be secured with password-protected access systems. Participants' study information will not be released outside of the study without the written permission of the participant, unless required by law.

Declaration of Interests
The principal researcher has no competing interests or conflicts of interest to declare.
Competing Interests: LM has received support from Pfizer Australia, Workers' Compensation Boards in Australia, North American and Europe, NOIgroup Australasia, Kaiser Permanente California, Results Physiotherapy, Agile Physiotherapy, the International Olympic Committee and the Port Adelaide Football Club. and receives royalties from the following books: Explain Pain; Explain Pain Handbook: Protectometer; Explain Pain Supercharged; Painful Yarns – Metaphors and Stories to Help Understand the Biology of Pain; the Graded Motor Imagery Handbook.

Access to Data
EK and GLM will have full access to the final trial dataset.

Ancillary and Post-Trial Care
Planning for the provision of ancillary care is not deemed necessary due to the low/negligible risk of this study.

Dissemination Policy
Trial results will be disseminated to key stakeholders within 3 months of study completion. This will include the communication of results to the Royal Adelaide Hospital (Research Ethics Committee, Research Governance, Spinal Unit, Physiotherapy Department), the University of South Australia (Research Ethics Committee) and the ANZCTR. Publication of the results of the feasibility study in a peer-reviewed journal is planned and will occur regardless of the study outcomes, and without restrictions. Study participants will be sent a link (via SMS) to the 'Body in Mind' website where publications arising from this study will be available.

The full study protocol will be made publicly available via the Open Science Framework website. There is no intention to make the complete data set publicly available for this feasibility study.

Discussion

The results of this feasibility study will inform the subsequent development of a definitive

randomised controlled trial of the GLITtER intervention. The reporting of this study will be carried out according to the ***CONSORT 2010 guideline*** for transparent and quality reporting of randomised pilot and feasibility trials (see www.consort-statement.org).

Date of Proposed Commencement and Duration

The Pilot Study will commence 2/3/2017. The clinical intervention phase will take less than 3 months. Follow-up data collection will be completed by September 2017.

Appendices

Informed Consent Materials
See Appendices 1 and 2

Questionnaires/Outcome Measures
See Appendices 3 – 7

Table 2. Trial Registration Data

Data Category	Information
Primary registry and trial identifying number	ANZCTR:
Date of registration in primary registry	February, 2017
Secondary identifying numbers	Not applicable
Source(s) of monetary or material support	Royal Adelaide Hospital Research Foundation; NHMRC
Primary sponsor	Royal Adelaide Hospital Research Foundation
Secondary sponsor(s)	University of South Australia
Contact for public queries	EK, Emma.Karran@mymail.unisa.edu.au
Contact for scientific queries	EK, Emma.Karran@mymail.unisa.edu.au; LM, Lorimer.Moseley@unisa.edu.au
Public title	GLITtER (Green Light Imaging Interpretation to Enhance Recovery): a psychoeducational intervention for adults with low back pain attending secondary care
Scientific title	A quasi-randomised, controlled, feasibility trial of GLITtER (Green Light Imaging Interpretation to Enhance Recovery) – a psychoeducational intervention for adults with low back pain attending secondary care
Countries of recruitment	Australia
Health condition(s) or problem(s) studied	Low back pain; Chronic low back pain
Intervention(s)	Intervention group: Integration of the GLITtER intervention into 'standard practice' consultation
	Control group: 'Standard practice' consultation
Key inclusion and exclusion criteria	Ages eligible for study: ≥18 years; Sexes eligible for study: all; Accepts healthy volunteers: no
	Inclusion criteria: adult patient (18-65 years), attending Spinal Assessment Clinic, low back pain (+/- leg symptoms), access to a lumbar CT or MRI scan (performed during the previous 6 months)
	Exclusion criteria: unable to speak and understand English, history of lumbar spine surgery
Study type	Quasi-randomised, controlled, feasibility trial
Data of first enrolment	March 2 2017
Target sample size	40 (20 intervention, 20 control)
Recruitment status	Will commence March 2017
Primary outcomes(s)	Determine the feasibility of recruitment and retention, assessment procedures, implementation and acceptability of the "GLITtER' intervention
Key secondary outcomes	Identify any modifications needed in the design of a larger effectiveness trial.
	Provide data (i.e. the standard deviation of the primary outcome measure: numeric rating scale score for pain at 3 month follow-up) to permit calculation of an appropriately powered sample for a subsequent randomised controlled trial

Government of South Australia
Central Northern Adelaide Health Service

Royal Adelaide Hospital
North Tce
ADELAIDE SA 5000
Tel +61 8 8222 4000
Fax +61 8 8222 5939
ABN 80 230 154 545
www.rah.sa.gov.au

Spinal Unit
Tel +61 8 8222 4466
Fax +61 8 8222 2480

INFORMATION SHEET for patients participating in the 'GLITtER'
(Green Light Imaging Interpretation to Enhance Recovery)
feasibility trial

It is well recognised worldwide that providing patients with reassurance is a very important part of managing low back pain. However, how best to reassure – particularly when details of back pain cause and prognosis are unclear – is poorly understood. One thing that seems common is that structural changes shown on back scans are often described in ways which can increase peoples' worries and make them very cautious about moving. This research is interested in whether more careful attention to how we explain scan results, can actually lead to improvements in recovery.

This is a research project and you do not have to be involved. If you do not wish to participate, your medical care will not be affected in any way. Also, you may withdraw from this project at any time after you have commenced.

If you agree to participate you will be asked to complete three questionnaires before your appointment today. Further to this, you may be asked to complete some additional forms on 2 occasions following your spinal outpatient clinic appointment. These can be mailed or emailed to you.

This project will not involve any foreseeable risks or adverse effects to you. Your name and personal results will be kept confidential at all times. The research will be conducted according to the NHMRC National Statement on Ethical Conduct in Human Research, 2007.

Contacts:

If you have any questions or concerns please contact the primary researchers: Emma Karran, Senior Physiotherapist, RAH, Tel. 8222 5334 or Professor Lorimer Moseley, Tel. 8302 2454 (during business hours). If you wish to speak to someone not directly involved in the study about your rights as a volunteer, or about the conduct of the study, you may contact the Chairperson, Research Ethics Committee, RAH on 8222 4139.

Government of South Australia
Central Northern Adelaide
Health Service

Royal Adelaide Hospital
North Tce
ADELAIDE SA 5000
Tel +61 8 8222 4000
Fax +61 8 8222 5939
ABN 80 230 154 545
www.rah.sa.gov.au

Spinal Unit
Tel +61 8 8222 4466
Fax +61 8 8222 2480

PARTICIPANT CONSENT FORM

Protocol Name: GLITtER (Green Light Imaging Interpretation to Enhance Recovery): a novel psycho-education intervention to reduce chronic pain. Phase 3 – Pilot Study

Investigators: Emma Karran, Lorimer Moseley, Yun-Hom Yau

1. The nature, purpose and risks of the research project have been explained to me. I understand them and I agree to take part.
2. I understand that I may not directly benefit from taking part in the study.
3. I understand that, while information gained during the study may be published, I will not be identified and my personal results will remain confidential.
4. I understand that I can withdraw from the study at any stage and that this will not affect my medical care, now or in the future.
5. I have had the opportunity to discuss taking part in this investigation with a family member or friend.
6. I give permission for the principal researcher to contact me via SMS or email on a maximum of 5 occasions over the following 3 months for follow-up related to my involvement in this study (if required).

Name: ______________________________

Email: ______________________________

Mobile: ______________________________

Signed: ______________________________ **Date:** / / 2017

INVESTIGATOR SIGNATURE ONLY:
I certify that I have explained the study to the patient and consider that he/she understands what is involved.

Signed: ______________________________ (Principal Investigator) Date: / /2017

Appendix 3 – Recommended Minimum Dataset (page 1)

Government of South Australia
Central Northern Adelaide
Health Service

Name: Date: / /17

1. How long has low-back pain been an ongoing problem for you?
   - ☐ Less than 1 month
   - ☐ 1-3 months
   - ☐ 3-6 months
   - ☐ 6 months – 1 year
   - ☐ 1-5 years
   - ☐ More than 5 years

2. How often has low-back pain been an ongoing problem over the past 6 months?
   - ☐ Every day or nearly every day in the past 6 months
   - ☐ At least half the days in the past 6 months
   - ☐ Less than half the days in the past 6 months

3. In the past 7 days, how would you rate the intensity of your pain on average? (circle a number)

0	1	2	3	4	5	6	7	8	9	10
No pain										Worst pain imaginable

4. Has back pain spread down your leg in the past 2 weeks?
   - ☐ Yes
   - ☐ No
   - ☐ Not sure

5. During the past 4 weeks, how much have you been bothered by.....

	Not bothered at all	Bothered a little	Bothered a lot
• Stomach pain	☐	☐	☐
• Pain in your arms, legs or joints (other than your back)	☐	☐	☐
• Headaches	☐	☐	☐
• Widespread pain or pain in most of your body	☐	☐	☐

6. Have you ever had a low-back operation?

☐ Yes, one operation
☐ Yes, more than one operation
☐ No

In the past 7 days....

7. How much has pain interfered with your day-to-day activities? (Circle a number)

0	1	2	3	4	5	6	7	8	9	10
No interference										Unable to carry on activities

	Not at all	A little	Somewhat	Quite a bit	Very much
**8.** How much did pain interfere with work around the home?	☐	☐	☐	☐	☐
**9.** How much did pain interfere with your ability to participate in social activities?	☐	☐	☐	☐	☐
**10.** How much did pain interfere with your household chores?	☐	☐	☐	☐	☐

11. Have you used any of the following treatments for your back pain? (Tick all that apply)

	Yes	No	Not sure
• Opioid painkillers (prescription medications such as Oxycodone, MS Contin, Codeine, Fentanyl, Duragesic, Oxycontin, Methadone, Tramadol)	☐	☐	☐
• Injections (such as epidural steroid injections, facet injections)	☐	☐	☐
• Exercise therapy	☐	☐	☐
• Psychological counselling, such as cognitive-behavioural therapy?	☐	☐	☐

The next two questions are for people who normally work outside the home:

12. I have been off work or unemployed for 1 month or more due to low back pain

☐ Agree ☐ Disagree ☐ Does not apply

13. I receive or have applied for disability or workers compensation benefits because I am unable to work due to low-back pain

☐ Agree ☐ Disagree ☐ Does not apply

	Without any difficulty	With a little difficulty	With some difficulty	With much difficulty	Unable to do
**14.** Are you able to do chores such as vacuuming or yard work?	☐	☐	☐	☐	☐
**15.** Are you able to go up and down stairs at a normal pace?	☐	☐	☐	☐	☐
**16.** Are you able to go for a walk of at least 15 minutes	☐	☐	☐	☐	☐
**17.** Are you able to run errands and shop?	☐	☐	☐	☐	☐

In the past 7 days....

	Never	Rarely	Sometimes	Often	Always
**18.** I felt worthless	☐	☐	☐	☐	☐
**19.** I felt helpless	☐	☐	☐	☐	☐
**20.** I felt depressed	☐	☐	☐	☐	☐
**21.** I felt hopeless	☐	☐	☐	☐	☐

In the past 7 days.....

	Very poor	Poor	Fair	Good	Very good
**22.** My sleep quality was	☐	☐	☐	☐	☐

	Not at All	A little bit	Somewhat	Quite a bit	Very much
**23.** My sleep was refreshing	☐	☐	☐	☐	☐
**24.** I had a problem with my sleep	☐	☐	☐	☐	☐
**25.** I had difficulty falling asleep	☐	☐	☐	☐	☐

26. It's not really safe for a person with my back problem to be physically active

☐ Agree ☐ Disagree

27. I feel that my back pain is terrible and it's never going to get any better

☐ Agree ☐ Disagree

28. Are you involved in a lawsuit or legal claim related to your back problem?

☐ Yes
☐ No
☐ Not sure

In the past year:

	Never	Rarely	Sometimes	Often
**29.** Have you been drunk or used drugs more than you meant to?	☐	☐	☐	☐
**30.** Have you felt you wanted or needed to down on your drinking or drug use?	☐	☐	☐	☐

31. Age: ______ years

32. Gender:

☐ Female ☐ Male

☐ Unknown ☐ Unspecified

33. Employment Status

- ☐ Working now
- ☐ Looking for work, unemployed
- ☐ Sick leave or maternity leave
- ☐ Disabled due to back pain, permanently or temporarily
- ☐ Disabled for reasons other than back pain
- ☐ Student
- ☐ Temporarily laid off
- ☐ Retired
- ☐ Keeping house
- ☐ Other, specify

34. Education Level

- ☐ Did not complete high school
- ☐ Completed high school
- ☐ Some further education; no degree
- ☐ Occupational/technical/vocational program
- ☐ Completed university degree
- ☐ Completed university post-graduate qualification

35. How would you describe your cigarette smoking?

- ☐ Never smoked
- ☐ Current smoker
- ☐ Used to smoke, but have now quit

36. Height: ________________ (self-reported)

Weight: ________________ (self-reported)

Appendix 4 – Tampa Scale for Kinaesiophobia-11

Government of South Australia
Central Northern Adelaide
Health Service

Name: Date: / /17

Please answer ALL statements and indicate whether you *strongly disagree*, *disagree*, *agree* or *strongly agree* with each statement by circling the appropriate number on the scale.

	Strongly Disagree	Disagree	Agree	Strongly Agree
1. I'm afraid that I might injury myself if I exercise	1	2	3	4
2. If I were to try to overcome it, my pain would increase	1	2	3	4
3. My body is telling me I have something dangerously wrong	1	2	3	4
4. My pain would probably be relieved if I were to exercise	1	2	3	4
5. People aren't taking my medical condition seriously enough	1	2	3	4
6. My accident has put my body at risk for the rest of my life	1	2	3	4
7. Pain always means I have injured my body	1	2	3	4
8. Just because something aggravates my pain does not mean it is dangerous	1	2	3	4
9. I am afraid that I might injure myself accidentally	1	2	3	4
10. Simply being careful that I do not make any unnecessary movements is the safest thing I can do to prevent my pain from worsening	1	2	3	4
11. I wouldn't have this much pain if there weren't something potentially dangerous going on in my body	1	2	3	4
12. Although my condition is painful, I would be better off if I were physically active	1	2	3	4
13. Pain lets me know when to stop exercising so that I don't injure myself	1	2	3	4
14. It's really not safe for a person with a condition like mine to be physically active	1	2	3	4
15. I can't do all the things normal people do because it's too easy for me to get injured	1	2	3	4
16. Even though something is causing me a lot of pain, I don't think it's actually dangerous	1	2	3	4
17. No one should have to exercise when he/she is in pain	1	2	3	4

Appendix 5 – Outcome Data: Pain and Disability NRS & Reassurance Questions (3)

Government of South Australia
Central Northern Adelaide
Health Service

Name: Date: / /17

1. In the past 7 days, how would you rate the intensity of your pain on average? (Circle a number)

0	1	2	3	4	5	6	7	8	9	10
**No pain**										**Worst pain imaginable**

2. In the past 7 days, how much has your pain interfered with your daily activities on average? (Circle a number)

0	1	2	3	4	5	6	7	8	9	10
**No interference**										**Unable to carry on activities**

3. Rate your belief in how likely it is that you will require an operation on your back? (circle a number)

0	1	2	3	4	5	6	7	8	9	10
**Not at all likely**										**Extremely likely**

4. How reassured are you that there is nothing seriously wrong with your back? (circle a number)

0	1	2	3	4	5	6	7	8	9	10
**Extremely reassured**										**Not at all reassured**

5. Do you think more tests or scans are needed at this stage? (circle) **Y / N**

Appendix 6 – Roland Morris Disability Questionnaire

Government of South Australia

Central Northern Adelaide
Health Service

Name: Date: / /17

When your back hurts, you may find it difficult to do some of the things you normally do.
Mark only the sentences that describe you **today.**

- ☐ I stay at home most of the time because of my back.
- ☐ I change position frequently to try to get my back comfortable.
- ☐ I walk more slowly than usual because of my back.
- ☐ Because of my back, I am not doing any jobs that I usually do around the house.
- ☐ Because of my back, I use a handrail to get upstairs.
- ☐ Because of my back, I lie down to rest more often.
- ☐ Because of my back, I have to hold on to something to get out of an easy chair.
- ☐ Because of my back, I try to get other people to do things for me.
- ☐ I get dressed more slowly than usual because of my back.
- ☐ I only stand up for short periods of time because of my back.
- ☐ Because of my back, I try not to bend or kneel down.
- ☐ I find it difficult to get out of a chair because of my back.
- ☐ My back is painful almost all of the time.
- ☐ I find it difficult to turn over in bed because of my back.
- ☐ My appetite is not very good because of my back.
- ☐ I have trouble putting on my socks (or stockings) because of the pain in my back.
- ☐ I can only walk short distances because of my back pain.
- ☐ I sleep less well because of my back.
- ☐ Because of my back pain, I get dressed with the help of someone else.
- ☐ I sit down for most of the day because of my back.
- ☐ I avoid heavy jobs around the house because of my back.
- ☐ Because of back pain, I am more irritable and bad tempered with people than usual.
- ☐ Because of my back, I go upstairs more slowly than usual.
- ☐ I stay in bed most of the time because of my back.

Appendix 7 – Participant Satisfaction Questionnaire

Government of South Australia
Central Northern Adelaide
Health Service

Name: Date: / /17

Please rate your responses to the following statements by ticking the appropriate box:

	Strongly disagree	Disagree	Agree	Strongly agree
1. The clinician who saw me in the Spinal Clinic knew what they were talking about				
2. The clinician used simple, clear language that I could easily understand				
3. The information on the handout was relevant to me				
4. The information on the handout was helpful				
5. It was good to get the follow-up text messages				
6. Overall, I was satisfied with the care I received from the Spinal Clinic				
7. The information I have received from the Spinal Clinic will assist my recovery				

8. Since my Spinal Clinic Appointment I have increased my activity level:
(tick the appropriate box) ☐ Not at all ☐ A little bit ☐ A lot

Please also provide us with some feedback regarding the links to further information:

WEEK 1: The Truth About Back Pain
Did you watch it? **Yes / No** Was it helpful? **Yes / No**

WEEK 2: Understanding Pain in Less than 5 Minutes
Did you watch it? **Yes / No** Was it helpful? **Yes / No**

WEEK 3: Exercise Right website
Did you look at it? **Yes / No** Was it helpful? **Yes / No**

WEEK 4: Managing Back Pain Podcast
Did you listen to it? **Yes / No** Was it helpful? **Yes / No**

Appendix 8 – Health Care Utilisation

Government of South Australia
Central Northern Adelaide
Health Service

Name: Date: / /17

Recent healthcare use:

1. In the past 2 months, **have you seen a general practitioner** for your back pain?

   Y / N If so, how many times? ____

2. In the past 2 months, **have you seen a physiotherapist** for your back pain?

   Y / N If so, how many times? ____

3. In the past 2 months, **have you seen a chiropractor, massage therapist or other health care professional** for your back pain?

   Y / N If so, how many times? ____

1. Chan A-W, Tetzlaff JM, Altman DG, Laupacis A, Gøtzsche PC, Krleža-Jerić K, Hróbjartsson A, Mann H, Dickersin K, Berlin JA: **SPIRIT 2013 statement: defining standard protocol items for clinical trials**. *Annals of internal medicine* 2013, **158**(3):200-207.
2. Koes BW, van Tulder M, Lin C-WC, Macedo LG, McAuley J, Maher C: **An updated overview of clinical guidelines for the management of non-specific low back pain in primary care**. *Eur Spine J* 2010, **19**(12):2075-2094.
3. Traeger AC, Hübscher M, Henschke N, Moseley GL, Lee H, McAuley JH: **Effect of Primary Care–Based Education on Reassurance in Patients With Acute Low Back Pain: Systematic Review and Meta-analysis**. *JAMA internal medicine* 2015.
4. Linton SJ, McCracken LM, Vlaeyen JW: **Reassurance: help or hinder in the treatment of pain**. *Pain* 2008, **134**(1):5-8.
5. Graves JM, Fulton-Kehoe D, Jarvik JG, Franklin GM: **Early imaging for acute low back pain: one-year health and disability outcomes among Washington State workers**. *Spine* 2012, **37**(18):1617-1627.
6. Webster BS, Cifuentes M: **Relationship of early magnetic resonance imaging for work-related acute low back pain with disability and medical utilization outcomes**. *Journal of Occupational and Environmental Medicine* 2010, **52**(9):900-907.
7. Brinjikji W, Luetmer P, Comstock B, Bresnahan B, Chen L, Deyo R, Halabi S, Turner J, Avins A, James K: **Systematic Literature Review of Imaging Features of Spinal Degeneration in Asymptomatic Populations**. *American Journal of Neuroradiology* 2014.
8. Steffens D, Hancock MJ, Maher CG, Williams C, Jensen TS, Latimer J: **Does magnetic resonance imaging predict future low back pain? A systematic review**. *Eur J Pain* 2014, **18**(6):755-765.
9. van Tulder MW, Assendelft WJ, Koes BW, Bouter LM: **Spinal radiographic findings and nonspecific low back pain: a systematic review of observational studies**. *Spine* 1997, **22**(4):427-434.
10. Hertzog MA: **Considerations in determining sample size for pilot studies**. *Research in nursing & health* 2008, **31**(2):180-191.
11. Deyo RA, Dworkin SF, Amtmann D, Andersson G, Borenstein D, Carragee E, Carrino J, Chou R, Cook K, DeLitto A *et al*: **Report of the NIH Task Force on Research Standards for Chronic Low Back Pain**. *The Journal of Pain* 2014, **15**(6):569-585.
12. Tkachuk GA, Harris CA: **Psychometric Properties of the Tampa Scale for Kinesiophobia-11 (TSK-11)**. *The Journal of Pain* 2012, **13**(10):970-977.
13. Jensen MP, Turner JA, Romano JM: **What is the maximum number of levels needed in pain intensity measurement?** *Pain* 1994, **58**(3):387-392.
14. Ornetti P, Dougados M, Paternotte S, Logeart I, Gossec L: **Validation of a numerical rating scale to assess functional impairment in hip and knee osteoarthritis: comparison with the WOMAC function scale**. *Annals of the rheumatic diseases* 2011, **70**(5):740-746.
15. Roland M, Morris R: **A study of the natural history of back pain: part I: development of a reliable and sensitive measure of disability in low-back pain**. *Spine* 1983, **8**(2):141-144.
16. Roland M, Fairbank J: **The Roland–Morris disability questionnaire and the Oswestry disability questionnaire**. *Spine* 2000, **25**(24):3115-3124.
